# A Longitudinal Study on Body Image Perception and Size among Italian Early Adolescents: Changes over Time and Discrepancies between Genders

**DOI:** 10.3390/nu16203439

**Published:** 2024-10-11

**Authors:** Emanuela Gualdi-Russo, Sabrina Masotti, Natascia Rinaldo, Federica De Luca, Stefania Toselli, Gianni Mazzoni, Simona Mandini, Luciana Zaccagni

**Affiliations:** 1 Department of Neuroscience and Rehabilitation, Faculty of Medicine, Pharmacy and Prevention, University of Ferrara, 44121 Ferrara, Italy; rnlnsc@unife.it (N.R.); federica.deluca@unife.it (F.D.L.); gianni.mazzoni@unife.it (G.M.); simona.mandini@unife.it (S.M.); luciana.zaccagni@unife.it (L.Z.); 2 Center for Exercise Science and Sports, University of Ferrara, 44123 Ferrara, Italy; 3 Department for Life Quality Studies, University of Bologna, 47921 Rimini, Italy; stefania.toselli@unibo.it

**Keywords:** perceived body image, perceived weight status, body dissatisfaction, body mass index, adolescents

## Abstract

Background/Objectives: The discrepancy between the current perceived body image (BI) and beauty ideals leads to dissatisfaction, which is believed to be common among adolescents. This study aimed to investigate the stability in BI perceptions and dissatisfaction during early adolescence. Another aim was to highlight differences in dissatisfaction according to Body Mass Index (BMI) and inconsistencies in weight status perception. Methods: Two hundred and nine participants (mean age at first survey: 11.33 ± 0.38 years) were enrolled in this longitudinal study with three years of follow-up. Data on size and BI perceptions were collected through individual interviews. Stature and weight were directly measured. Results: Findings indicated significant changes over three years in anthropometric traits but not in weight status prevalence or BI perception and dissatisfaction, except for the ideal figure in males and weight control in females. The results also indicated a significant difference in BI perception and dissatisfaction by BMI categories. Self-reported body measurements were found to be unreliable with a weak-to-moderate agreement between self-perceived and actual weight status. Conclusions: BI perception appears quite stable across the three years considered. Dissatisfaction is similar in both genders, although a tendency toward different gender aesthetic ideals is already appreciable in early adolescence. We suggest that the lower frequency of normal-weight adolescents compared with peers in previous studies is attributable to the effects of the recent pandemic. Given the growing dissatisfaction with increasing BMI and misinterpretations of weight status, school actions to promote a healthy lifestyle and positive BI should be undertaken.

## 1. Introduction

Body image (BI) perception corresponds to a subjective image of the body, which may disregard its actual appearance. Indeed, it is a composite construct that includes feelings, evaluations, and attitudes related to the body [1,2]. This perception is also influenced by feedback from other people [3]. When there are negative cognitions and feelings toward body image, we speak of body image dissatisfaction [4].

The importance of BI perception is so great that it can affect the individual’s physical and mental health status. Judging one’s appearance depends heavily on the ideal body image in society [4], which associates the ideal of beauty with thinness in the Western world [5,6]. BI dissatisfaction, defined as individuals’ detrimental and subjective mental representation of body appearance, is widespread among adolescents [7]. At the developmental stage of adolescence, when major mental, physical, and social changes occur, the perception of BI is generally exacerbated with a tendency to develop concerns about it. At this stage, adolescents are particularly susceptible to the comments and opinions of peers and adults about their bodies. They also tend to abandon family as a reference to focus on friends and peers, such that the perceptions and evaluations of these reference groups condition their self-concept and self-esteem [8]. Dissatisfaction with BI, due to discrepancies between reality, perception of one’s body, and desired ideal, is often determined in adolescents, deeply affecting their self-esteem [9].

Adolescent self-esteem is conditioned by the perception of BI and the judgment given by peers according to fixed canons that, following sexual dimorphism, impose thin and toned bodies for females and muscular and strong bodies for males [5,6]. Unlike in some non-Western countries [10], what happens in Western countries is that these body ideals end up increasing the risk of psychological and physical illnesses often accompanied by eating disorders. An eating disorder is a severe mental health condition that can develop from a poor BI and can have physical health consequences with possible cardiovascular, endocrine, gastrointestinal, and fertility complications. These eating disorders include anorexia, bulimia, and binge eating disorder [11].

During the pubertal transition, adolescents increase in stature and weight and change their body structure and composition [12,13]. In this period of pubertal development, differences between males and females increase in physical appearance and psychological profile, with various health implications [14]. Along with sexual dimorphism, there is growing awareness among adolescents of gender differences that are reflected in physical appearance. It is mainly girls who manifest problems regarding their BI and body weight, so much so that more than 70% of female adolescents wish to change their body weight or shape [15]. The onset of puberty can result, especially in girls, in a series of transformations that drive them away from the ideal of thinness, exposing them to teasing from peers [16]. These aspects have great relevance as body dissatisfaction in adolescence is believed to be a significant risk factor leading to eating disorders [17,18]. Epidemiological studies conducted in Italy have shown that a high percentage of adolescents are at risk for eating disorders [19] with increased underweight or overweight status [20].

Studies in the literature assessing changes in body dissatisfaction during adolescence have led to inconsistent results, which is probably because of the different developmental ages and contexts analyzed [21,22]. Cross-sectional studies cannot answer the questions, which are still open, concerning the possible changes in BI perception and dissatisfaction from early adolescence when growth and development occur.

Therefore, using a longitudinal design, our main purpose was to examine possible changes in body perception and dissatisfaction in girls and boys across middle school years. We hypothesized that girls are characterized by greater and increasing body dissatisfaction with age than boys because they wish to comply with an ideal of thinness.

Another purpose was to explore body dissatisfaction by BMI over three years and any inconsistencies in participants’ perceptions of their weight status. In this regard, we hypothesized that dissatisfaction is greater in unhealthy weight categories and that there is a proper perception of weight status.

## 2. Materials and Methods

### 2.1. Participants and Procedures

This longitudinal study is part of more extensive ongoing research on the physical and mental health of children and early adolescents in northern Italy [23,24,25,26,27].

The Bioethics Committee of the University of Bologna approved the study procedures (approval code no. 2.18).

Before this longitudinal study was undertaken, we determined the minimum sample size required to test the primary study hypothesis by the G*Power statistical program (version 3.1.9.6; Universitat Kiel, Kiel, Germany) by ANOVA for repeated measures (3 measurements) in 2 groups (males and females) for 95% power, medium effect size, and 0.05 significance level, resulting in 44 participants.

To this end, we invited all students from a middle school in Ferrara (Emilia-Romagna region, Northern Italy) to participate. The school (chosen on a convenience basis) cooperated by sending an informational letter to all students’ families and requesting written, signed parental consent. Twenty students without signed consent from their parents were excluded from the surveys. The final sample included 209 schoolchildren (121 boys and 88 girls) who had signed consent and agreed to participate. Anonymity and confidentiality were assured to all participants, who had the right to withdraw from the study at any time.

The longitudinal data of each schoolchild completing grades 6–8 were collected at a 1-year interval from November 2020 to November 2023. The mean age in the first survey for participants was 11.33, with a standard deviation (SD) of 0.38.

Due to careful monitoring, no missing values were found in the body image perception questions or anthropometric measurements collected.

### 2.2. Anthropometric Survey

A simple proxy for body composition in these early adolescents was obtained through Body Mass Index (BMI), that is, the weight-to-stature ratio (kg/m^2^). Through this index, the students’ weight status can be assessed according to Cole cutoffs [28,29] as underweight (UW), normal weight (NW), overweight (OW), or obese (O). To this end, the same operator directly measured stature to the nearest 0.1 cm with an anthropometer (Magnimeter, Raven Equipment Ltd., Dunmow, Essex, UK) and weight to the nearest 0.5 kg with a mechanical scale (SECA, Basel, Switzerland) on participants dressed in light clothing, following standardized anthropometric methods [30,31,32].

### 2.3. Size and Body Image Perception

Before the anthropometric survey, students’ face-to-face interviews were conducted within a separate setting allowing for greater privacy and concentration.

After some basic socio-demographic questions, we tested the participants regarding awareness of body size by asking the following: “How tall are you?”, “How much do you weigh?” (for these first two questions, given the age of the participants, the answer “I don’t know” was also scheduled), “Which weight status do you think best represents you?” (choice: underweight, normal weight, overweight/obese). We tested participants for possible dissatisfaction and weight interventions by asking the following: “Do you control your weight?” (choice: never, sometimes, often, always), “Have you tried to lose weight in the last year?” (choice: never, sometimes, often, always) and “If yes, please indicate in what manner” (choice: reducing food, increasing physical activity).

Moreover, to assess body image perception and dissatisfaction, we employed the scale of Childress et al. [33] consisting of eight drawings of female or male figures obtained by child adaptation of an adult figure scale [34]. These eight silhouettes of equal stature were sorted separately by gender in an incremental manner from a skinny figure (silhouette 1) to an obese one (silhouette 8). On this scale, participants had to mark which figure best represented their actual body image and which represented their ideal figure. This evaluation was repeated longitudinally in all surveying years. BI dissatisfaction (Feel minus Ideal Discrepancy, FID) was assessed by subtracting the selected ideal figure from the actual feel figure [35,36]. Dissatisfaction increases as the absolute value of the score rises: positive values indicate that the dimensions perceived as real are greater than ideal ones; negative values indicate that the dimensions perceived as real are smaller than ideal ones. No dissatisfaction exists when the figure perceived as real equals the ideal figure (FID score = 0).

Aware of the difference between the terms sex and gender [37], we use the former when related only to biological characteristics and the latter in all other cases (e.g., when related to cultural, psychological, or behavioral characteristics or when these factors are variously intertwined with biological factors).

### 2.4. Statistical Analysis

We calculated absolute and relative frequencies for each qualitative variable (questionnaire responses and weight status categories) and means and SD for each quantitative variable (anthropometric and BI perception traits) separately by gender. The normality of quantitative variables was assessed through the Kolmogorov–Smirnov test.

The chi-squared test was used to detect whether the observed frequencies differed from the expected ones in comparisons by gender and time. To test the effect of sex on normally distributed quantitative variables at times T0, T1, and T2, we used ANOVA for repeated measurements. A nonparametric Friedman rank-sum test was applied to variables not normally distributed, such as variables related to BI perception. In addition, in these cases, we performed a gender comparison separately at times T0, T1, and T2 with the Wilcoxon test.

A paired samples t-test was applied to compare measured anthropometric traits with the ones reported by participants.

The main effects of perceived BI variables with the different BMI categories were analyzed by the Kruskal–Wallis one-way analysis of variance with rank ANOVA.

To assess participants’ ability to place themselves in the correct weight status category, we calculated Cohen’s kappa coefficient, a measure of agreement for qualitative variables.

All the analyses were conducted using Statistica for Windows, Version 11.0 (StatSoft Srl, Tulsa, OK, USA), and R software version 4.4.1 for Windows.

*p* values less than 0.05 were assumed to be statistically significant.

## 3. Results

### 3.1. Changes in Anthropometric Traits, Weight Status, BI Perception

Table 1 shows the anthropometric characteristics of participants, measured directly by us and self-reported by participants during the three years (Time 0, 1, 2), and statistical comparisons.

The ANOVA for repeated measures shows a significant effect of time and a significant interaction of time and sex on five traits (stature and weight, BMI; stature and weight, self-reported). Specifically, concerning interaction, it appears that participants increased in dimensions over the three years, but the male sex showed greater increases than the female sex, especially in stature. Even time had a statistically significant effect on BMI, as calculated from self-reported data. The main effect of sex on traits was not significant.

The comparison between direct and self-reported measurements revealed the low reliability of the reported values (pairwise *t*-test): the self-reported weight was significantly lower than that measured in boys in all three surveys (T0 *p* < 0.0001; T1 *p* = 0.0014; T2 *p* = 0.0124) and in girls at T0 (*p* = 0.0002) and T1 (*p* = 0.0009); self-reported stature was significantly higher than that measured only in girls at T2 (*p* = 0.0155).

The second part of Table 1 shows the prevalence of weight status in the sample in three years. Normal-weight participants were prevalent in both sexes in all three surveys. In boys, there was a tendency for an increase in underweight and overweight from T0 to T2 and a concomitant decrease in obesity. Conversely, in girls, underweight tended to decrease from T0 to T1/T2 while obesity tended to increase. However, chi-square comparisons show that changes from time T0 to T2 did not achieve statistical significance in males or females. In comparisons between the two sexes for the prevalence of different weight status categories, no significant differences were found at T0, T1, and T2 (*p* > 0.05).

Table 2 shows the BI perception variables and answers to questions submitted to participants.

The perception of BI did not differ among time T0, T1, and T2 except for the ideal figure in males (tending to a more robust ideal figure in T2). In separate gender comparisons at each time, there was a significant difference concerning feel figures (T0: W = 4229, *p*-value = 0.009; T1: W = 4340, *p*-value = 0.03; T2: W = 4227, *p*-value = 0.008) and ideal ones (T0: W = 3165.5, *p*-value < 0.0001; T1: W = 3617.5, *p*-value < 0.0001; T2: W = 2959.5, *p*-value < 0.0001). In particular, girls perceived themselves as thinner than their male peers and liked slimmer body figures more than boys. This trend was reflected in the FD scores showing the highest dissatisfaction in the females even without reaching the statistical significance level (*p* > 0.05).

The perception of weight status did not show significant changes in the three surveys or comparisons between genders in each survey (*p* > 0.05).

Considering the answer to the question about participant weight control, most boys and girls in sixth and seventh grades said they had tried to control their weight “sometimes”.

Fifty percent of eighth-grade girls answered “never”. However, more than 20% of girls of the same age said they did it often or always. Notably, this percentage had doubled or tripled from previous classes. Thus, significant changes were observed in females over the three years compared to males. Gender comparison was found to have significant differences only in T2 (*p* < 0.05).

Through the other question, no significant change was observed over the three years regarding weight loss attempts in the two genders. We found that girls tried to lose weight more than boys, but the differences between the genders were close to statistical significance only at T2 (0.10 < *p* > 0.05). Among participants who reported trying to lose weight, most of them took action by both reducing food and increasing physical activity (Males: 40.4% in T0, 45.8% in T1, and 43.9% in T2; Females: 36.4% in T0, 39.4% in T1, and 42.2% in T2); a part of them merely reduced food (Males: 8.5% in T0, 18.7% in T1, and 22% in T2; Females: 27.3% in T0, 30.3% in T1, and 28.9% in T2) and another increased physical activity (Males: 29.8% in T0, 22.9% in T1, and 24.4% in T2; Females: 27.3% in T0, 39.4% in T1, and 17.8% in T2).

### 3.2. BI Perception and Weight Status

Table 3 compares the relevant BI perception variables in T0, T1, and T2 according to the BMI category in boys and girls. All the BI variables were significantly different among weight status categories except for the ideal figure in males (in T0, T1, and T2) and females (in T0 and T1). The mean feel figure and, to a lesser extent, the ideal figure tended to increase with BMI in both genders over the three times considered. The same trend was observed for the FID. Negative FID values characterized underweight male participants (only in T1 for underweight females), indicating their propensity toward more robust ideal figures than those with whom they identified (feel figures). In contrast, the overweight/obese participants showed positive FID values, demonstrating their leaner ideals compared to the feel figures that characterized them.

Finally, we calculated Cohen’s kappa coefficient to assess the agreement between the current weight status assessed by BMI (through direct anthropometric measurements) and the perceived weight status of the participant (through the answer to the question: *Which weight status do you think best represents you?*) (Table 4).

Based on Cohen’s kappa, agreement strength appeared weak-moderate in both genders and for all repetitions [38,39]. The coefficient trend over the three years indicates a gradually increasing awareness of one’s weight status as age increases, particularly in girls. It is also evident that there was a tendency for underweight participants of both genders to overestimate their weight status and underestimate it in overweight/obese participants.

## 4. Discussion

The adolescent stage constitutes, on the one hand, a period of intense growth and remarkable morpho-metric changes with the appearance of secondary sexual characters and, on the other hand, a sensitive period for mental health because of the need to adapt to a new appearance of one’s body [40]. This is compounded by the beauty ideals imposed especially by the Western world, also due to social media, that exalt muscular development in males and an ectomorphic, thin body in females [5,6]. When adolescents perceive that their bodies differ from these ideals, dissatisfaction with their body image is often determined. In particular, psychological factors and pressures from peers, family, and the media to conform to these socially prescribed body ideals contribute to the onset and persistence of body dissatisfaction, explaining the associations between BI and weight status [41].

This longitudinal study examined anthropometric and body image perception changes in early adolescents over three years to assess any trends over time and any gender differences.

As expected, significant changes were observed in most anthropometric traits and indices with repeated measurements over time, as well as sex and the interaction between these two factors. Regarding weight status, we found no significant differences in category frequencies over the three years, nor were there any significant differences between sexes. Participants also had a clear awareness of their size (stature and weight): the reliability of the reported values most likely depends on whether participants remember the measures routinely taken by the family doctor. Moreover, slightly lower self-reported values were probably due to measurements taken previously in growing subjects during medical checkups.

Comparing the weight status of the examined sample with data from the recent literature for Italian adolescents, we found a lower percentage of normal-weight boys and, especially, girls compared to data collected longitudinally in the Emilia-Romagna region five or more years earlier ([23]: 11 years of age: 68.8% in males and 82.4% in females; 12 years of age: 76.2% in males and 85.7% in females; 13 years of age: 77.8% in males and 82.9% in females) and the national average data referred to the 11 and 13 years of age and collected cross-sectionally two years earlier ([42]: 11 years of age: 71.6% in males and 80.3% in females; 13 years of age: 73.1% in males and 83.1% in females). Also noteworthy is that there was twice the frequency of underweight in the male subsample examined and similar frequencies in the female subsample compared with national data ([42]: 11 years of age: 3.3% in males and 4.3% in females; 13 years of age: 1.9% in males and 2.5% in females); in the regional study [23], underweight was present with very low frequencies in 11- and 12-year-old girls (11 years of age: 1.5%; 12 years: 1.5%) while no underweight was found among boys and 13-year-old girls. The overweight/obese participants of our sample also showed remarkable differences with generally higher frequencies, particularly in girls (>10%), compared with regional data ([23]: 11 years of age: 31.3% in males and 16.2% in females; 12 years of age: 23.8% in males and 12.8% in females; 13 years of age: 22.2% in males and 17.1% in females) and national data ([42]: 11 years of age: 25.1% in males and 15.4% in females; 13 years of age: 25.0% in males and 14.4% in females). In summary, the extreme categories (underweight, overweight/obese) generally show higher frequencies than other Italian samples previously examined. A possible explanation for this pattern, in addition to the observed worldwide trend in the prevalence of obesity and overweight in children and adolescents [43], is that the sample we examined was adversely affected by the COVID-19 outbreak and the virus containment measures, foremost of which was the lockdown with the interruption of school activities in attendance, sports, and social activities. This situation is recognized in the literature as being generally responsible for the rise in BMI [44,45,46] due to the intake of unhealthy foods, lifestyle changes, and poor engagement in physical activities [47]. The negative influences of lockdown on eating behaviors have been shown in the Italian population [47] and other populations around the world [48,49]. While this hypothesis is acceptable to justify the origin of the phenomenon, it is interesting to note that the higher category of weight status (examined longitudinally) remained constant over time also in the post-COVID period (the state of emergency ended on 31 March 2022) [50], as comparisons in later surveys of our study show. In this regard, we cannot rule out the possibility that the bad habits acquired during the COVID isolation have persisted. Moreover, in contrast to the increased prevalence of overweight/obese described in several populations as an effect of the pandemic, we also found an increased prevalence of underweight in the examined sample of adolescents. This trend, probably attributable to the negative impact of the restrictions on mental health and possibly inadequate nutrition, was also found in other recent European studies on Polish adolescents aged 11–15 years [51] and German primary school children [52].

In this situation, observing any trends in the perception of BI may be particularly interesting. Among the quantitative variables related to BI perception, feel figures and FID in both genders and the ideal figures in females, on the other hand, remained stable over time. Meanwhile, the ideal figures varied significantly over time in boys: the progressive preference for a more robust body ideal is due to the likely misconception that the selected ideal figures correspond to greater muscularity [41,53]. The ideal figures chosen by girls were significantly thinner than in boys at all ages, and there was a tendency for a higher FID score in girls than in boys. This trend corresponds to what has been reported in the literature for adults regarding the greater BI dissatisfaction of women, the thinner BI ideal in females, and the more muscular BI ideal in males [54,55,56,57]. In particular, under the influence of Western media, the ideal male body is generally presented as muscular, athletic, and lean [5,56] and the female body thin, tall, and ectomorphic [5,56,58]. The longitudinal study allows us to appreciate how these trends are nuanced during early adolescence and evolve slowly over the period under consideration. Although the degree of BI dissatisfaction (FID) was higher in girls than in boys approaching +1 (the dissatisfaction threshold according to Mendo–Lazaro, 17 [59]), this did not reach statistical significance in the gender comparisons in the likeness of what was found in a cross-sectional study of a sample of Italian children aged 5 to 12 years examined in 2022–2023 [25]. In early adolescents, as observed in preadolescents [59], dissatisfaction with BI is similar in both genders, and the typical gender-differentiated pattern evidenced at later ages is not clearly visible [5] also if we can already distinguish the predominant ideal gender aesthetics.

According to the answers provided to the questions presented to the participants, in addition to a decline in weight control over the three years (significantly in females), the results show that most participants never attempted to lose weight, keeping this trend stable over the three years, consistent with their young age. Among those who made attempt to reduce body weight, the most common method was simultaneous diet control and exercise, followed by food reduction alone and, ultimately, by increased physical activity alone.

As previously reported, an analysis of the perceived vs. ideal figure shows that both genders prefer a leaner body. This trend is even more evident when considering the sample divided into weight status categories: FID increases with BMI. In particular, moving from underweight to overweight, boys’ dissatisfaction scores rose to a greater extent but decreased with age (T0: +2.65; T1: +2.50; T2: +2.22) compared to those of girls (T0: +1.82; T1: +2.31; T2: +1.07). This analysis also reveals negative scores across the three surveys in underweight males who were found to be dissatisfied with their status and with a desire for more robustness. In contrast, scores varied slightly between complete satisfaction and mild dissatisfaction at the three times in underweight girls. In this regard, it should be pointed out that overweight/obese adolescents are often the object of teasing by their peers [60]. According to recent research conducted in Italy [61], these individuals showed not only the highest ratings of verbal victimization related directly to body shape but they were frequently subjected to being physically attacked and barred from playing sports and participating in group activities. Social relationships are generally more problematic and worse as weight status (actual or self-rated) increases.

This study demonstrates the need to use directly measured anthropometric measures because self-referred ones are not adequately reliable (especially weight). Moreover, the consistency between the weight status perceived and the actual one established by BMI was low: we observed a weak-moderate agreement between current weight status and self-perceived weight status, which means that the reliable data in the examined sample varied from 15% to 63% [39]. These interrater reliability levels can be considered low in terms of the possible need for health care. In particular, the health risks of some participants in the extreme weight categories should be emphasized: a few underweight boys and girls perceived themselves to be overweight/obese, and a few overweight/obese boys and girls perceived themselves to be underweight. These misperceptions can result, if left untreated, in BI or eating disorders [59]. Moreover, about half of the overweight/obese participants perceived their weight status as normal during the three surveys: underestimating one’s weight status results in the continuation of any obesogenic behaviors, with the consequent risk of increasing overweight/obesity status [56,62]. School and local health services policy should safeguard adolescents’ physical and mental health by involving families in an integrated effort aimed at a healthy lifestyle. This will also be effective against various chronic health and social problems, including peer bullying victimization.

Our study had various strengths. The most relevant of these strengths concerns the study’s longitudinal design: we examined the same sample of early adolescents over three years. The second strength concerns the direct detection of anthropometric traits involved in the BMI calculation by the same expert operator. The third point concerns the face-to-face interaction between the adolescent and the operator in detecting the participants’ BI perception and awareness of body size. This allowed participants to focus on the questions without fear of being judged by peers. As for the study’s weaknesses, we did not examine the participants’ eating and living habits or the families’ socioeconomic conditions, although these may affect their nutritional status [63], nor did we assess their ethnic origins [64]. Moreover, a comparison with other studies is difficult because of the small number of research with a longitudinal design in early adolescents during and after COVID periods and because of the use of different possible methods of BI assessment (different silhouette scales, questionnaires). Finally, the standardized silhouette scale of Childress et al. (1993) [33] that we applied did not allow for the assessment of ideal muscularity in males. To the best of our knowledge, however, there are currently no scales for assessing muscularity in early adolescents, unlike adults [57,65].

We expect that further research can provide a longer perspective on longitudinal variability in weight status and BI perception in cohorts of adolescents who have experienced the effects of lockdown from COVID-19 in different parts of the world. Research would also be desirable to arrive at silhouette scales that distinguish between muscularity and fatness for a better interpretation of adolescent BI.

## 5. Conclusions

Our study demonstrates the stability of early adolescents’ BI perception: only the body ideal changes over time in boys toward greater robustness. In females, a tendency toward greater dissatisfaction and a preference for leaner body ideals are already present at this young age. However, while conforming to gender ideals, preadolescent males and females show similar levels of BI dissatisfaction, although this has been shown to increase with BMI.

Concerning the weight status of the examined sample, we found an increase in the prevalence of both overweight/obesity and underweight compared with previous studies. These weight patterns can be attributed to lingering consequences of psychological and living conditions established during restrictions by the COVID-19 pandemic. The self-perception of weight status is not entirely satisfactory, showing a tendency to overestimation in underweight adolescents and underestimation in overweight/obese adolescents.

We expect that, especially in the school setting, actions will be taken to promote a healthy lifestyle and BI. In particular, it would be necessary to implement educational efforts at the school level to equip students, teachers, and parents with valuable tools such as guidelines for healthy living (from nutrition to physical activity) to control unhealthy weight status, negative BI, and their consequences. At the same time, early detection of BI perception issues should be encouraged as it can be critical in identifying at-risk adolescents.

## Figures and Tables

**Table 1:** Anthropometric characteristics and comparisons by sex with ANOVA for repeated measures (total n = 209; males n = 121; females n = 88) and with chi-square for weight status frequencies over time.

Variables	Boys			Girls			*ANOVA*		
	T0	T1	T2	T0	T1	T2	*Sex*	*Measures*	Sex * *Measures*
	*Mean* *(SD)*	*Mean* *(SD)*	*Mean* *(SD)*	*Mean* *(SD)*	*Mean* *(SD)*	*Mean* *(SD)*	*F* *(p)*	*F* *(p)*	*F* *(p)*
Stature (cm)	150.25 (8.48)	156.99 (9.09)	164.00 (9.05)	151.31 (7.07)	156.08 (6.31)	158.17 (5.95)	3.04 (0.08)	1209.63 (<0.0001)	142.87 (<0.0001)
Weight (kg)	45.48 (11.49)	50.97 (12.28)	56.58 (13.21)	45.58 (9.49)	50.56 (10.05)	53.47 (10.16)	0.54 (0.46)	491.77 (<0.0001)	16.14 (<0.0001)
BMI (kg/m^2^)	19.94 (3.78)	20.53 (3.92)	20.89 (3.89)	19.82 (3.37)	20.72 (3.72)	21.34 (3.65)	0.12 (0.73)	69.68 (<0.0001)	3.83 (0.02)
Stature (cm) self-reported	150.08 ^1^ (11.22)	155.95 (11.96)	163.75 (12.04)	151.01 ^2^ (8.09)	155.88 (6.11)	159.08 (6.36)	1.62 (0.20)	216.33 (<0.0001)	18.49 (<0.0001)
Weight (kg) self-reported	43.17 ^1^ (10.22)	49.45 (11.93)	55.44 (12.77)	43.26 ^2^ (7.91)	48.27 (8.95)	52.62 (8.95)	1.70 (0.19)	229.59 (<0.0001)	6.98 (0.001)
	**Boys**						**Girls**		
	**T0**	**T1**	**T2**	*Chi-square*			**T0**	**T1**	**T2**
	*N (%)*	*N (%)*	*N (%)*	*p-value*		*p-value*	*N (%)*	*N (%)*	*N (%)*
*Weight status*				>0.05		>0.05			
UW	8 (6.6)	9 (7.4)	10 (8.3)				5 (5.7)	2 (2.3)	2 (2.3)
NW	76 (62.8)	73 (60.3)	72 (59.5)				55 (62.5)	60 (68.2)	56 (63.6)
OW	25 (20.7)	29 (24.0)	31 (25.6)				24 (27.3)	21 (23.9)	24 (27.3)
O	12 (9.9)	10 (8.3)	8 (6.6)				4 (4.5)	5 (5.7)	6 (6.8)

Note: The “I don’t know” response to the self-reported dimensions resulted in a reduction of the sample as follows: ^1^ n = 113 in Stature, n = 109 in Weight; ^2^ n = 83 in Stature, n= 81 in Weight.

**Table 2:** BI perception and dissatisfaction by gender and weight control.

BI Variables	Boys			*Friedman* *Rank-Sum Test*		Girls		
	T0	T1	T2			T0	T1	T2
	*Mean (SD)*	*Mean (SD)*	*Mean (SD)*	*p-Value*	*p-Value*	*Mean (SD)*	*Mean (SD)*	*Mean (SD)*
Feel Figure	4.55 (1.30)	4.59 (1.33)	4.77 (1.26)	0.099	0.17	4.09 (1.21)	4.16 (1.28)	4.31 (1.24)
Ideal Figure	3.90 (0.89)	3.89 (0.94)	4.13 (0.68)	0.0008	0.15	3.28 (0.79)	3.42(0.83)	3.45 (0.86)
FID (score)	0.65 (1.25)	0.69 (1.31)	0.64 (1.22)	0.73	0.47	0.81 (1.19)	0.74 (1.20)	0.85 (1.15)
	**Boys**			*Chi-square*		**Girls**		
	**T0**	**T1**	**T2**			**T0**	**T1**	**T2**
**Questions**	*N (%)*	*N (%)*	*N (%)*	*p-value*	*p-value*	*N (%)*	*N (%)*	*N (%)*
*Which weight status do you think best represents you?*				>0.05	>0.05			
-UW	10 (8.3)	13 (10.7)	13 (10.7)			7 (8.0)	7 (8.0)	5 (5.7)
-NW	80 (66.1)	78 (64.5)	81 (66.9)			60 (68.2)	60 (68.2)	58 (65.9)
-OW/O	31 (25.6)	30 (24.8)	27 (22.3)			21 (23.9)	21 (23.9)	25 (28.4)
*Do you control your weight?*				>0.05	<0.001			
-never	36 (29.8)	33 (27.3)	47 (38.8)			26 (29.5)	34 (38.6)	44 (50.0)
-sometimes	68 (56.2)	69 (57.0)	57 (47.1)			56 (63.6)	41 (46.6)	25 (28.4)
-often	13 (10.7)	12 (9.9)	15 (12.4)			5 (5.7)	8 (9.1)	14 (15.9)
-always	4 (3.3)	7 (5.8)	2 (1.7)			1 (1.1)	5 (5.7)	5 (5.7)
*Have you tried to lose weight in the last year?*				>0.05	>0.05			
-never	74 (61.2)	73 (60.3)	80 (66.1)			55 (62.5)	50 (56.8)	43 (48.9)
-sometimes	36 (29.8)	34 (28.1)	28 (23.1)			26 (29.5)	25 (28.4)	34 (38.6)
-often	7 (5.8)	8 (6.6)	12 (9.9)			5 (5.7)	11 (12.5)	11 (12.5)
-always	4 (3.3)	6 (5.0)	1 (0.8)			2 (2.3)	2 (2.3)	0 (0)

**Table 3:** BI perceptions by BMI categories in boys and girls.

BI Variables	BMI Categories								
	T0			T1			T2		
***Boys***	UW *Mean (SD)*	NW *Mean (SD)*	OW/O *Mean* *(SD)*	UW *Mean (SD)*	NW *Mean (SD)*	OW/O *Mean* *(SD)*	UW *Mean (SD)*	NW *Mean (SD)*	OW/O *Mean* *(SD)*
Feel Figure	2.75 (0.89)	4.16 (0.99)	5.76 (0.95)	3.00 (1.12)	4.22 (1.02)	5.66 (81.15)	3.30 (0.95)	4.39 (0.90)	5.85 (1.14)
*Kruskal–Wallis*	*H* 54.81		*p* <0.0001	*H* 41.45		*p* <0.0001	*H* 47.45		*p* <0.0001
Ideal Figure	3.75 (1.04)	3.82 (0.76)	4.11 (1.07)	3.78 (0.83)	3.90 (0.92)	3.89 (1.01)	3.90 (0.74)	4.11 (0.64)	4.23 (0.74)
*Kruskal–Wallis*	*H* 2.91		*p* 0.23	*H* 0.41		*p* 0.81	*H* 2.48		*p* 0.29
FID (score)	−1.00 (1.20)	0.34 (0.90)	1.65 (1.18)	−0.78 (0.97)	0.32 (0.93)	1.72 (1.30)	−0.60 (0.70)	0.28 (0.88)	1.62 (1.23)
*Kruskal–Wallis*	*H* 42.00		*p* <0.0001	*H* 44.35		*p* <0.0001	*H* 45.61		*p* <0.0001
***Girls***	UW *Mean (SD)*	NW *Mean (SD)*	OW/O *Mean* *(SD)*	UW *Mean (SD)*	NW *Mean (SD)*	OW/O *Mean* *(SD)*	UW *Mean (SD)*	NW *Mean (SD)*	OW/O *Mean* *(SD)*
Feel Figure	3.60 (1.82)	3.65 (0.82)	5.04 (1.23)	2.00 (0.00)	3.72 (1.01)	5.35 (0.98)	3.00 (1.41)	3.82 (0.90)	5.30 (1.21)
*Kruskal–Wallis*	*H* 22.76		*p* <0.0001	*H* 37.46		*p* <0.0001	*H* 27.35		*p* <0.0001
Ideal Figure	3.60 (1.34)	3.29 (0.66)	3.21 (0.92)	2.50 (0.71)	3.40 (0.79)	3.54 (0.90)	2.50 (0.71)	3.34 (0.75)	3.73 (0.98)
*Kruskal–Wallis*	*H* 0.31		*p* 0.86	*H* 3.26		*p* 0.20	*H* 6.94		*p* 0.03
FID (score)	0.00 (1.58)	0.36 (0.73)	1.82 (1.25)	−0.50 (0.71)	0.32 (0.79)	1.81 (1.33)	0.50 (2.12)	0.48 (0.83)	1.57 (1.30)
*Kruskal–Wallis*	*H* 32.03		*p* <0.0001	*H* 31.41		*p* <0.0001	*H* 18.57		*p* 0.0001

**Table 4:** Consistency between current weight status and self-perceived weight status.

Current Weight Status	Self-Perceived Weight Status								
	T0			T1			T2		
**Boys**	UW	NW	OW/O	UW	NW	OW/O	UW	NW	OW/O
UW	3	3	2	5	4	1	5	4	1
NW	7	62	7	7	57	8	7	61	4
OW/O	0	15	22	1	17	21	1	16	22
*Kappa*	*0.439*			*0.405*			*0.476*		
**Girls**	UW	NW	OW/O	UW	NW	OW/O	UW	NW	OW/O
UW	2	1	2	1	1	0	1	1	1
NW	5	46	4	5	50	6	4	47	5
OW/O	0	13	15	1	9	15	0	10	19
*Kappa*	*0.424*			*0.454*			*0.508*		

## Data Availability

Data are available upon request due to ethical restrictions regarding participants’ privacy. Requests for the data may be sent to the corresponding author.
